# A Doctor Needing Reconstruction

**Published:** 1874-03

**Authors:** 


DR. E. S. GAILLARD, Editor of the *Richmond* and *Louisville Medical Journal*, has our sincere sympathy in his recent bereavement in the loss of a *supporter*, we copy the following letter as showing the loss which he has sustained.

GOSHEN HILL, Union Co., S. C.

DR. E. S. GAILLARD:

Sir:—I can not support a Journal that advocates a social reunion with the northern section of the Medical Association under the circumstances.

Very respectfully, etc.,

G—— D——, M. D.

Evidently Dr. G—— D——, who seems ashamed to sign his name in full, needs a little wholesome reconstruction. We did feel sorry for the people of South Carolina, but if they are all like the Doctor, we must "under the circumstances" withdraw our sympathy.

